# Predictors of physical activity among women in Bojnourd, north east of Iran: Pender’s health promotion model

**DOI:** 10.1186/s13690-021-00698-x

**Published:** 2021-10-14

**Authors:** Seyedeh Belin Tavakoly Sany, Mohammad Vahedian Shahroodi, Zahra Hosseini Khaboshan, Arezoo Orooji, Habibollah Esmaeily, Alireza Jafari, Mohammad Tajfard

**Affiliations:** 1 Department of Health Education and Health Promotion, Faculty of Health, Mashhad University of Medical Sciences, Mashhad, Iran; 2 Social Determinants of Health Research Center, Mashhad University of Medical Sciences, Mashhad, Iran; 3 Department of Epidemiology and Biostatistics, Faculty of Health sciences, Mashhad University of Medical Sciences, Mashhad, Iran; 4 Department of Health Education and Health Promotion, School of Health, Social Development and Health Promotion Research Center, Gonabad University of Medical Sciences, Gonabad, Iran

**Keywords:** Women, Physical activity, Health promotion model, Health behavior, Health education, Public health,

## Abstract

**Background:**

This study examined how socio-demographic characteristics constructs derived from the health promotion model (HPM) influence the level of physical activity (PA) women in Bojnourd, North East of Iran.

**Method:**

This cross-sectional study has been carried out through multi-stage sampling design on 356 women aged 18–60 years living in Iran. Data was collected through reliable and valid questionnaire survey women who were selected from their homes.

**Results:**

Most of participants (53.40%) had a low level of physical activity behaviors with minimal physical activity (PA) scores. Using regression analysis showed that 23.22% of the total variance in PA behaviors was predicted by socio-demographic variables, whilst 40.81% of the variance in PA behaviors was predicted by constructs from the HPM. The results from Path modeling indicated that prior behavior, interpersonal influences, perceived self-efficacy, perceived barriers and commitment to PA, were significant predictors for PA behaviors with 86.93% of total effects on PA, whereas, activity-related affect and situational influences had no significant effect on the PA behaviors.

**Conclusions:**

We found that constructs derived from the HPM are determinants of PA among Iranian women and may be important in developing educational intervention programs to facilitate a physically active lifestyle in this population.

**Supplementary Information:**

The online version contains supplementary material available at 10.1186/s13690-021-00698-x.

## Background

Promoting regular physical activity (PA) is a key public health goal in both developed and developing countries [1–3]. Changes in PA pattern (e.g., sedentary life styles, passive modes of transport and hence less physical activity) are a major cause of mortality and morbidity for women globally [4–6]. Despite considerable efforts being made to improve PA within some populations, inadequate PA remains prevalent in many regions, particularly in the Middle East Countries (29%), America (32%), and Eastern Mediterranean (31%) [7]. Across all these regions, women were less active and had a higher prevalence of obesity then men and their pattern of healthy lifestyle are main concern [6].

There remains wide variation across countries in the health organization of services available to address the needs of health behaviors. This could be due to the impact of a socio-cultural, environment and economic factors that influence an individual’s health status [8, 9]. In Iran, health status and clinical characteristics have been improved greatly over the last two decades because of extending public health preventive service and health-system performance through the establishment of comprehensive insurance plan and an extensive healthcare delivery network [10, 11]. Although more national programs have been conducted to improve health status in Iran, large numbers of women suffer from disease processes that are associated with insufficient PA [12–16].

The World Health Organization (WHO) has reported that approximately 33.5% of adults aged 18 years and over in Iran (24.15% men and 42.90% woman) are not sufficiently active to achieve health benefits [14]. Notably, among racial/gender groups, Iranian woman have very high levels of inactivity, in part because of cultural factors associated with attitudes to PA that differ from Western cultural attitudes to PA [1, 10]. For example, Islamic women in Iran choose to wear the ‘hijab’ (a modest dress code), to participate in gender segregation, and to have controlled access to their PA space [14]. However, more efforts have been made to modify PA behaviors in Iran, the effect of social norms, cultural standards, family and relative attitudes toward PA have not been studied systematically, especially with respect to women population [12–14]. It is still unclear what/how potential determinates can affect/impact women’s commitment and self-efficacy to regular PA [13].

A variety of health promotion models have been developed to evaluate the psychometric properties and health promotion behaviors at the population level [17–20]. The Pender’s health promotion model (HPM) is a theoretical perspective to identify determinants that might affect an individual’s commitment to healthy behaviors [21, 22]. The Pender’s HPM is derived from the social cognitive theory and consists of eight possible constructs (Figure S1). Individual characteristics and prior experiences is the first construct of the HPM model to evaluate prior related behavior of the individuals in the past, and innate factors that influence health promotion behaviors [17].

In the HPM, behavior-specific cognition and affects are evaluated using the following constructs: perceived barriers, benefits, self-efficacy, and activity-related affect. These constructs are the main target to perceive the positive consequences, hurdles, beliefs, and intentions to carry out a particular health behavior for most HPM research. This increases cognitive skills by helping individuals to make new choices effectively and enables them to make changes to their own behavior [18, 21, 22]. Perceived self-efficacy is a central construct that evaluates the personal capability and self-confidence in the development of desired outcomes or health behaviors. The situational and interpersonal influences are important constructs examining the effect of social (family and friend support and interference) and environmental support (home and neighborhood) on health behavior [23, 24].

Commitment to planning and behavioral outcomes is assessed to examine the individuals’ belief, intention and health decision-making regarding behaviors end point. Several studies have reported that the potential constructs of HPM provide a comprehensive model to assist researchers to understand the complex bio-psychosocial processes and major determinants of healthy behaviors as a basis for behavioral counseling to promote health-promoting behavior [23, 24]. Further, this model has been successfully implemented in numerous intervention program to modify health behaviors such as taking responsibility of health, nutrition, exercise, stress management, and interpersonal support [22].

In this study, we used the Pender’s HPM to identify the factors associated with a high level of PA, which may in turn affect healthy lifestyle in women. Furthermore, performing this type of research in Iran, as an Islamic country, may be particularly crucial when attempting to evaluate the effects of barriers, and intrapersonal and social factors on PA behaviors [6]. The primary objectives of this study was to: 1) explore how socio-demographic characteristics, prior behavior, behaviors-specific cognition, and perceived social support, are associated with the level PA women undertake; 2) examine whether constructs from Pender’s HPM are determinants of PA among women.

## Methods

### Sample size determination and sampling

The required sample size was estimated using the following formula [25]:

$$ \mathrm{Sample}\ \mathrm{size}={Z}_{1-\frac{\alpha }{2}}^2P\left(1-P\right)/{d}^2 $$

Where, Z is standard normal value that considered at 5% type 1 error (*P* < 0.05) it is 1.96, P, is expected proportion in population based on previous studies or pilot studies. According to previously published studies actual proportion of PA may not be more than 38% [14]. After adjusting for non-response of 10%, 398 women were enrolled as sample size. Finally, 365 women were included in the data analysis because 33 women did not answer to all items in questionnaires.

We used the multi-stage sampling design to survey the female population sample in Bojnourd, Razavi Khorasan province, Iran. This city is the Metropolis of North East of Iran. The main reason for multistage cluster sampling in the present study was cost efficiency as well as it need less time for listing and implementation, and can help reduce costs of large-scale study for sampling. We divided the city into 20 regions (or clusters) based the presence of primary health care center (or health-house). The first-stage of sampling selected 3 domains using probability sampling weighted by the number of family (consisting of two parents and their children) from each of these regions (total 60 domains). The number of households in each region was derived from municipality annual report. Second-stage sampling included a simple random sample to select one block from each domain. Subsequently, we used systematic sampling to select 10 families from each block. Overall, 600 families were enrolled and, for each family, a woman aged 18–60 years was selected. Briefly, 600 women were selected, of whom 205 (24.22%) did not meet the inclusion criteria, refused to participate (30, 5%) because of traveling and lack of time) and. Finally, 365 (60.80%) women were included in the data analysis, who completed all questionnaires.

Participants were included if they (a) able to complete all relevant questions contained within the administered questionnaires; (b) were age 18–60 years; (c) lived in Bojnourd during the previous 6 months; (d) could read and speak their native language (Persian). Participants were excluded if they (a) were unable to give informed consent (55 women); (b) were unwilling to participate (95 women); (c) had suffered mental disturbance (8 women), visual impairment (15 women), upper/lower limb disability and walking abnormality (33 women). All women completed the consent form and completed study instruments in a written format.

### Measures

We used the framework of Pender’s Health Promotion Model (HPM) to design the questionnaire [26]. Prior to collecting the data, we studied the relevant literature relating to the HPM model [9, 13] and translated all the constructs of the HPM model into Persian based on international guidelines for cross-cultural adaptation (CCA) [13] (Table S1). The questionnaire was given to an expert panel affiliated to Mashhad University of Medical Sciences comprising ten specialists: in exercise, diet, behavioral psychology and health education who reviewed the relevance and necessity of all items in order to quantify the content validity ratio (CVR) and content validity index (CVI). The average CVR and CVI for the PA behaviors questionnaire were 0.98 and 0.92, which were acceptable in present study.

We conducted a test re-test reliability to ensure the internal validity and that the measurements obtained were valid and stable over time in the same population. Therefore, we asked 30 independent women to fill questionnaire over two time to assess participants’ knowledge about the purpose of the test and degree of relationship between the data points. Then, we corrected minor wording errors and questions that were ambiguous were changed to improve clarity of the items. Among all constructs, a correlation coefficient was ranged from 0.71 to 0.84, which is acceptable reliability. Then, we evaluated the face validity and reliability (Cronbach’s alpha) regarding repeatability and internal consistency of questionnaire [27]. The overall Cronbach’s alpha of PA questionnaire was 0.85, indicating a strong internal consistency of the questionnaire’s criterion.

The questionnaire was completed by 365 eligible women. Interviews and self-assessment questionnaires were undertaken at the women’s homes or at a convenient public place, and lasted 60–120 min. All eligible women were informed about the aim of the study, including all specific terms related to cognitive, emotional, and behavioral aspects were also explained and interpreted to ensure the eligible participant would understand the meaning of wordings and decreased distractions and negative emotional states. At each interview, all women were asked about their socio-demographic characteristics (gender, age, employment status, income, marital status, educational level, and number of children) and were then asked to complete the PA questionnaire. Further, we measured their weight and height to compute body mass index (BMI). All clinical information included comorbid conditions (heart failure, diabetes, and coronary artery diseases) and mental disturbance was extracted from electronic health records (EHRs). This allows access to an individual’s health history including clinics, hospitals, pharmacies, doctors, and laboratories.

### Methodological design of the HPM

Items on the questionnaire were designed based on the nine constructs of the Pender’s model to examine physical activity behaviors [21] (Figure S1). Physical activity is defined as “bodily movement caused by skeletal muscles that results in increased energy expenditure including incidental movement and purposeful exercise” [4]. Individual characteristics and experiences were the first constructs of HPM model to be evaluated (Table S2, Table S3); prior related behaviors and innate factors that predict future behavior. We used a self-administered Iranian version of International Physical Activity Questionnaire (IPAQ-L) to examine the prior behaviors regarding physical activities over the previous week [28]. In this questionnaire, 24 validated short questions on regular physical activities (frequency and duration of leisure, work, and commuting and household/yard activities during the last 7 days) were included to evaluate learning experiences of individuals that influence PA level. Cronbach’s alpha for this scale was 0.83. The scale for perceived self-efficacy contained 8 items to examine the strength of the participants’ confidence regarding their ability to engage in regular PA despite various conflicting situations (e.g., the degree of confidence to conduct PA even if they were tired?) [22, 23]. Items in this construct ranged from 0 (no confidence) to 4 (great confidence). The Cronbach’s alpha for this scale was 0.91. Activity-related affect was measured using five items to examine effect of positive emotions or feeling states occurring during and prior PA (e.g., how much they agree with PA behavior enjoyment or affect), which were rated on a 5-point Likert scale ranging from 0 (completely disagree) to 4 (completely agree) [21, 29]. Cronbach’s alpha of this scale was 0.81.

We used 20 items to assess the perceived barriers (family obligations, lack of time and skills, health problems) and benefit (improving their lifestyle, prevention of chronic diseases, decreased nervousness and staying young). Participants were asked to rate how much they agreed with the perceived barriers (e.g., I might not perform regular PA if I didn’t have enough time) and benefits (e.g., a reason I perform regular PA, I feel exercise improves my physical appearance/ or decrease my nervousness). Items in these constructs ranged from 0 (completely disagree) to 4 (completely agree) and the Cronbach’s alpha for this scale was 0.87 and 0.89 for perceived benefit and barriers, respectively. Commitment to a plan contains 2 items to examine their intention to carry out PA (e.g., do you have regular schedule to perform PA?) with a two - point Likert scale ranging from 0 (No) to 1 (yes) [21, 23]. Cronbach’s alpha of this scale was 0.89. Interpersonal influences was measured using eight items to examine the frequency of social supports, modeling, expectations of others regarding women engagement in PA and norms given by family members and friends who encouraged them for engaging in PA (e.g., how often does your husband/ parents changes their schedule so they can exercise with you?; or my parents/husband expects me to exercise) [21, 29]. This construct was rated using a five - point Likert scale ranging from 0 (never) to 4 (always) with alpha of 0.94. We measured situational influences (walking environment in home and neighborhood, policies, economic condition, weather, inaccessible exercise facilities) using six items (e.g., I think it is easy for me to access exercise facilities/ in home/neighborhood, I think that cost of exercise is reasonable in my city) [22, 23]. This construct was rated from 0 (completely disagree) to 4 (completely agree). Behavioral outcome contained 8 items to examine the participants’ belief and health decision-making regarding PA end point (e.g., I consider regular schedule to perform PA during week, I manage my time to engage in regular PA despite various conflicting situations) [21, 22]. This constructs was rated from 0 (never) to 4 (always). Cronbach’s alpha of this scale was 0.89 (Table 1).

**Table 1:** Summary of all constructs from HPM assessed via self-reported questionnaire in this study

Constructs	Objectives	Statement
Individual characteristics and experiences *α* ^*b*^ = .83, *n*^a^ = 24	To clarify prior related behavior that influence PA	Participants were asked to indicate: regular physical activates (the weekly frequency and duration of sport, leisure, setting time, Job, transport, and household, caring family/yard activities during the last 7 days) using short-IPAQ-L [13].
Perceived self-efficacy *α* = .91, *n* = 8	To examine the strength of the participants’ confidence regarding their ability to engage in regular PA despite various conflicting situations	Participants were asked to rate their self-confidence and capability in different situations: e.g., how much degree of confidence do you have to conduct physical activity even if you were tired?, rated from 0 (no confidence) to 4 (great confidence) [21, 22].
Activity-related affect *α* = .81, *n* = 5	To examine effect of positive emotions or feeling states occurring during and prior PA	Participants were asked to select how much they agree with PA behavior enjoyment or affect: e.g., I enjoy exercising, rated from 0 (completely disagree) to 4 (completely agree) [21, 22].
Perceived Barriers *α* = .91 *α* = 10	To evaluate barriers perceived as preventing them from performing physical activity	Participants were asked to rate how much they agree with the perceived barriers such as family obligations, lack of knowledge and skills, health problems: e.g., I might not perform regular PA if I didn’t have enough time, or I don’t have a good place to exercise; rated from 0 (completely disagree) to 4 (completely agree) [21, 22].
Perceived Benefits *α* = .87, *n* = 10	To evaluate levels of agreement regarding perceptions and commitments about expected benefits of regular PA	Participants were asked to rate how much they agree with perceived benefits such as improving their lifestyle, prevention of chronic diseases, decreased nervousness and staying young: e.g., a reason I perform regular PA, I feel exercise improves my physical appearance/ or decrease my nervousness, 0 (completely disagree) to 4 (completely agree) [13, 21, 22].
Commitment to a plan *α* = .89, *n* = 2	To examine their intention to carry out PA	Participants were asked to clarify their commitment statues: e.g., do you have regular schedule to perform physical activity? or do you have commitment to your regular schedule regarding PA?, rated from 0 (No) to 1 (yes) [13].
Interpersonal influences *α* = .94, *n* = 8	To examine the frequency of social supports, modeling and norms given by family members and friends who encouraged them for engaging in PA; To examine vicarious learning through observing others person engaged in PA; To assess expectations of others regarding women engagement in PA	Participants are rated the frequency of perceived support: e.g., how often does your husband/ parents changes their schedule so they can exercise with you?; or My friends exercise with little effort or My parents/husband expects me to exercise; rated 0 (never) 4 (always) [13, 21, 22].
Situational influences *α* = .79, *n* = 6	To evaluate the level of participant’s perceptions about different situation that influences their appropriate PA	Participants were asked to rate how much they agree with the situational influences such as (walking environment in home and neighborhood), policies, economic condition, weather, inaccessible exercise facilities: e.g., I think it is easy for me to access exercise facilities/ in home/neighborhood, I think that cost of exercise is reasonable in my city. Rated from 0 (completely disagree) to 4 (completely agree) [21, 22].
Behavioral outcomes *α* = .89, *n* = 8	To examine the participants’ belief and health decision-making regarding PA end point	Participants were asked to select how much they agree with regular PA: e.g., I consider regular schedule to perform PA during week, I manage my time to engage in regular PA despite various conflicting situations, rated 0(never) to 4 (always) [21, 22].

^a^number of question

^b^Cronbach’s alpha

### Statistical analyses

SPSS 16 (Chicago, Illinois) was used to determine the descriptive statistics for all variables. Descriptive analyses were used to describe socio-demographic characteristics, as well as the level of PA behavior outcomes and HPMs’ constructs. A series of multiple analytical models (hierarchical multiple regression and Path analysis) were built with the objective of examining whether constructs from Pender’s HPM are empirically influences PA behaviors, which in turn influences their health lifestyle among Iranian women.

We used hierarchical multiple regression analysis to examine the association between the independent variables; prior behaviors, behavior-specific cognition, situational and interpersonal factors against the dependent variable PA behavior after controlling for the effects of age, marital status, education level, employment status, income, BMI, number of children and home ownership. The variables were entered into a regression model based on theoretical and logical considerations in present study. This allowed estimates of the contributions of the independent variables to be computed [25].

Path analysis was used to test the HPM on its constructs and how they were related and the directionality of significant relationships with PA behaviors outcomes [30]. The data were analyzed by maximum likelihood estimation (ML) of Path analysis using AMOS for Windows version 8.80. The ratio of the chi-square (x^2^) statistic to its degree of freedom, with a value less than 5 indicating an acceptable fit. A number of fit indices were recommended under intense scrutiny to assess model fit. These were the root mean square error of approximation (RMSEA), the adjusted goodness-of-fit index (AGFI), goodness of fit (GFI), the comparative fit index (CFI), Tucker-Lewis index (TLI) and the standardized root mean residual (SRMR). The model was considered to be a good fit if the RMSEA ≤0.08, SRMR < 0.05, AGFI > 0.80 and other indices (NFI, GFI, TLI) more than 0.90 [31, 32].

## Results

### Descriptive statistics and association analysis

In total, 365 women completed the study. Their mean (SD) BMI and age were 25.57 ± 6.56 kg/m^2^ and 30.05 ± 9.42 years, respectively. Most of the eligible participants were housewives (84.9%), with a moderate-income family (60.81%), married (83%), diploma (26.30%) or bachelor’s degree (27.48%) level of education with 3 or 4 children (60.86%) as well as have minimal PA (53%) (Table 2).

**Table 2:** Subject demographic and clinical characteristics

Characteristics *(n = 365)*	Sub-characteristics	Values
Age*, years, mean ± SD*	Range: 18–60	30.05 ± 9.4
BMI^d^ *kg/m*^*2*^*, mean ± SD*	Range: 17.5–33.6	25.5 ± 6.5
Menopause status, *n (%)*	Yes	51 (13.97)
	No	314 (86.03)
Education level*, n (%)*	Illiterate	57 (15.60)
	< High school	96 (26.81)
	> High school	14 (3.80)
	Diploma	96 (26.31)
	Higher education	100 (27.41)
Employment status, *n (%)*	Un-Employed	310 (84.90)
	Employed	55 (15.1)
Marital status, *n (%)*	Married	303 (83)
	Single	42 (11.52)
	Widow	20 (5.41)
Income, *n (%)*	Low	136 (373)
	Moderate	222 (60.80)
	High	7 (1.92)
Number of children, *n (%)*	1–2	23 (22.51)
	3	36 (35.31)
	4	26 (25.50)
	5	10 (9.80)
	5–10	7 (6.93)
Home ownership status*, n (%)*	Owner	166 (45.50)
	Tenant	199 (54.55)
History Comorbid conditions, *n (%)*	Yes	75 (20.56)
	No	290 (79.53)
PA Category based on IPAQ-L, *MET, n (%)*	Inactive^a^	90 (24.66)
	Minimal active^b^	195 (53.42)
	High Active^c^	80 (21.91)

±: Showing mean score (standard deviation); n: number of eligible participants

^a^Not located at criteria for active or minimally active

^b^Minimal active Include any of following criteria: 1) 3 or more days of vigorous activity for at least 20 min per day; 2) 5 or more days of moderate-intensity activity or walking for at least 30 min per day; 3) 5 or more days of any combination of walking, moderate-intensity or vigorous intensity activities achieving a minimum of at least 600 MET (metabolic equivalent)-min/week

^c^High active cover either of the following condition: 1) participating in 7 or more days of any combination of vigorous-intensity PA, moderate-intensity or walking, achieving a at least 3000 MET-m/ week or 2) attending in vigorous intensity PA a minimum 3 days achieving at least 1500 MET-m/week

^d^BMI was classified as underweight (< 18.5 kg/m^2^), normal (18.5–24.9), overweight (25–29.9) and obese (> 30) [33]

According to the regression model, step1, socio-demographic variables accounted for 23.22% of the variance of PA behaviors. In step 2, the individual characteristics and experiences (prior behaviors) was added to step 2 resulted in 8.90% increase of the explained variance of PA behaviors. In step 3, the inclusion of behavior-specific cognitions, interpersonal influences, situational influences, and commitment to PA were also added to the model 3 resulting in a 31.90% increase of the explained variance of PA behaviors. The final regression model showed that 63.71% of the total variance in PA behaviors were predicted by BMI, education level, number of children, employment statues, family income, prior behaviors, self-efficacy, interpersonal influences, and commitment to PA, with positive associations found between PA and education level, family income, prior behaviors, self-efficacy, and commitment to PA, and a negative association found between BMI, number of children, employment statues and interpersonal influences (Table 3). On the other hand, in this population, were not found to be significantly associated to engagement in PA.

**Table 3:** Summary of hierarchical regression analysis for variables predicting physical activity behavior among women population

Determinants *(n = 365)*	Step 1		Step 2		Step 3	
	*Beta*	*t*	*Beta*	*t*	*Beta*	*t*
Constant		3.47***		2.86**		1.5*
Mean Age	−.11	−1.40	−.09	− 1.35	−.06	−.1.12
BMI	−.19	−2.41*	−.17	− 2.02*	−.15	− 1.81
Menopause status *(1, yes; 0, no)*	−.09	−1.32	−.06	− 1.11	−.04	−.80
Education Level (*1, Illiterate; 5, higher education)*	.24	2.86**	.22	2.42**	.21	2.23**
Employment Status *(1, un-employed; 2, employed)*	- .22	−2.76**	−.18	− 2.12*	−.16	− 2.01*
**Marital status**						
*Married*	.10	1.420	.07	1.07	.06	.92
*Single*	.04	0.58	0.03	.48	.026	.37
*Widow*	.03	0.51	.024	.32	.018	.28
Income (*1, low; 3, high)*	.20	2.56*	.17	2.28*	.16	2.14*
Number of children(*1, 1–2; 5,5–10)*	−.26	−2.92**	−.23	− 2.48**	−.20	− 2.13*
Home ownership status *(1, tenant; 2, owner)*	.03	0.52	.03	.51	.02	.32
History Comorbid conditions *(1, yes; 0, no)*	−.07	−1.12	−.05	−1.02	−.03	−.72
Prior Behaviors	–	–	.17	2.33*	.16	2.17*
Perceived self-efficacy	–	–	–	–	.18	2.3*
Activity-related affect	–	–	–	–	.004	1.71
Perceived Barriers/benefits	–	–	–	–	−.10	−2.12*
Interpersonal Influences	–	–	–	–	−.42	5.61***
Situational Influences	–	–	–	–	−.014	−0.65
Commitment	–	–	–	–	.29	3.82**
R^2^	.23	–	.31	–	.63	–
R^2^ Changes	.22	–	.28	–	.31	–
F	4.21	–	5.25	–	7.02	–
P	< 0.001	–	< 0.001	–	< 0.001	–

The β values are called regression weights and are computed in a way that minimizes the sum of squared; Dependent Variable: physical activity Behavior; *** *p* < 0.001, ** *p* < 0.01, * *p* < 0.05

### Empirical testing of HPM

**Fig. 1:**
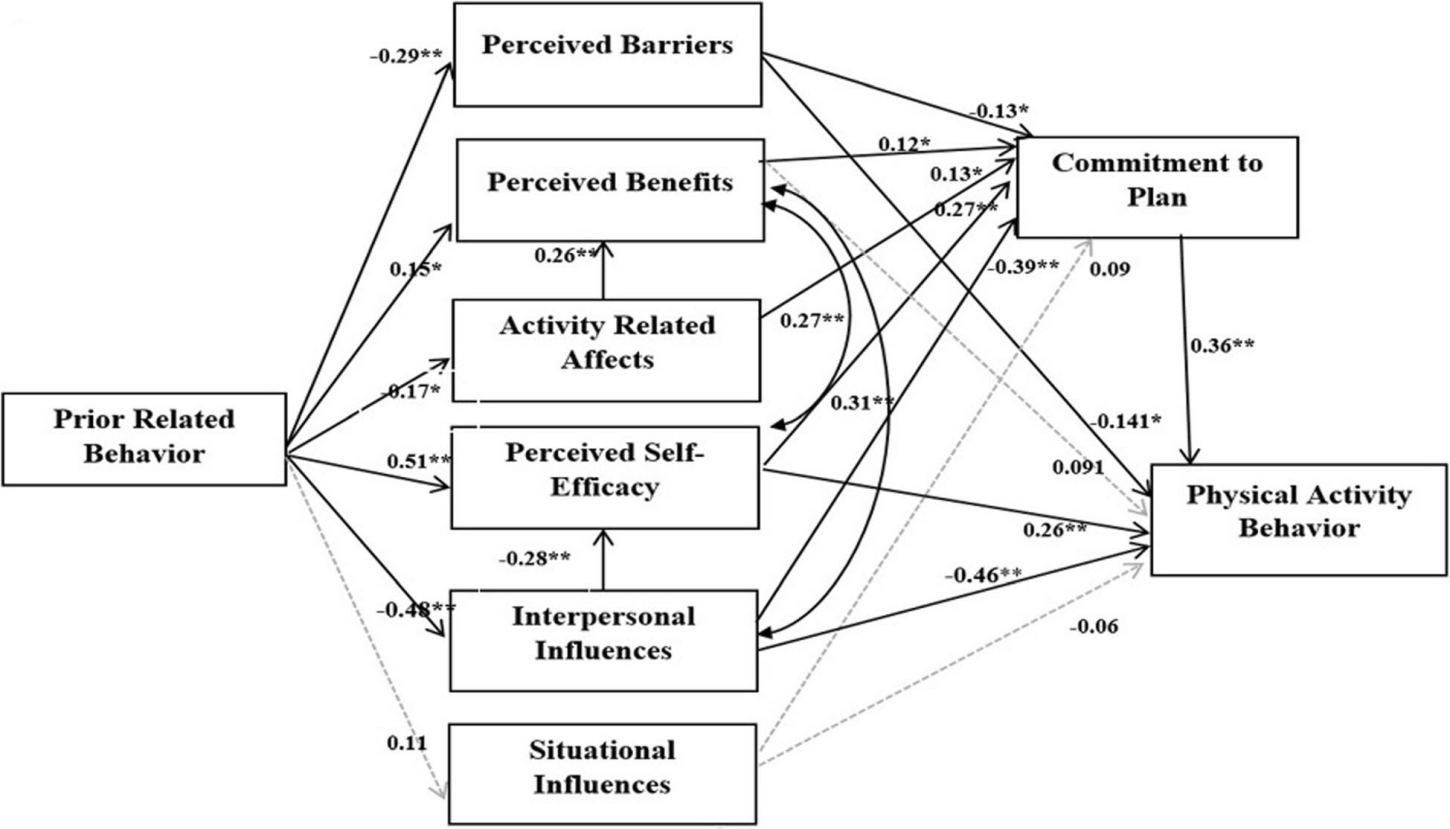
A path model of influences of priors’ behaviors, behavioral specific cognitions and competing demands on physical activity (as health promotion behaviors) among Iranian women (adapted from the Pender’s Health Promotion Model). Narrow line is non-significant effect and bold line is significant effects (*p < 0.05, **p < 0.01)

In this study, we run Path analysis with modification because the univariate statistical analysis showed that the distribution of the variables was moderately non-normal. Several study reported that estimation of maximum likelihood estimation, used in Path analysis, has been observed to be sensitive to the violation of normality [31, 32]. Therefore, we used logarithmic transformations to modify model (Table S4). The identification of the modified Path model implies that the hypothesized direction of effects among the model constructs showed acceptable fit by the data (Model identification indexes: χ^2^ = 3.60, *n* = 365); RMSEA = 0.072; AGFI = 0.84, GFI = 0.94; CFI = 0.92, TLI = 0.90). The Path model accounted for 46% of the variance in Iranian women’ participation in PA. The Path model for PA was tested (Fig. 1) using the following pathways: (a) Prior behavior directly influence participation in PA, (b) Prior behaviors indirectly influence participation in PA through physical activity-related cognitions and commitment to plan, (c) physical activity related to behavior-specific cognitions directly and indirectly influence participation in physical activity, and (d) commitment to plan directly influence participation in PA.

The results suggested that, there was no direct effect of prior behaviors on physical activity, whereas, there was a significant indirect effect of prior behaviors through enhancing behavioral-specific cognitions and commitment to plan. Testing the behavior-specific cognitions showed that perceived self-efficacy had a significant positive effect (standardized path coefficient (SPC) = 0.372, *p* < 0.01) on promoting PA, while interpersonal influences (SPC = − 0.63, *p* < 0.01) and perceived behaviors (SPC = − 0.179, *p* < 0.05) had the largest negative and significant influence on PA. It was evident that the effects of perceived self-efficacy and interpersonal influences on physical activity occurred mainly through the direct pathways. In addition, a significant positive effect of commitment to plan on PA was found (SPC = − 0.36, *p* < 0.01). In summary, among HPMs’ constructs, prior behavior, interpersonal influences, perceived self-efficacy, perceived barriers and commitment to plan were significant predictors for PA behaviors than others construct of Pender’s Model; their total effects accounted for 86.9% of total effects after estimation. Among these constructs, interpersonal influences were the strongest predictor, indicating about 28.5% of the total effects on PA (Fig. 1 and Table 4).

**Table 4:** Standardized total effects, direct, and indirect effects of HPMs’ constructs on physical activity behaviors derived from the Path

Determinants or Predictors	Causal Effect		
	Direct pathways	Indirect pathway	Total effects
Through priors behaviors	0	0.38	0.38
Through perceived barriers	−0.14	− 0.03	− 0.17
Through perceived benefits	0.09	0.05	0.14
Through activity related Affects	0.007	0.04	0.05
Through perceived self-efficacy	0.26	0.11	0.37
Through interpersonal influences	−0.46	−0.17	−0.63
Through situational influences	−0.06	−0.03	− 0.09
Through commitment to PA	0.36		0.36
Through total causal effect	1.37	0.83	2.20
Percantage effects	1.379/2.209 = **62.42%**	0.83/2.209 = **37.57%**	

## Discussion

Our finding showed that 77% of participants were not undertaking the recommended amounts of PA (PA > 30 min for 3 days per week), which is significantly lower than the standard level. American college of sports medicine and the American heart association recommend that individuals need to perform “30 to 60 minutes moderate-intensity aerobic physical activity for a minimum of three days each week to prevent chronic diseases” [34]. Iranian women undertake low levels of PA that that is consistent with other similar studies in Iran [11, 13]. We found that education and family income exert a statistically significant influence on PA behavior, which suggests that individuals with a high educational attainment and income had a greater intention to perform healthy behaviors in comparison with other women. This results is in line with existing empirical work that showed education level and family income notably explaining high variance or having a significant contribution to PA [9, 10]. Our findings showed that women who were an employee or had more than 2 children tend to have poor health promoting behaviors. Specific findings from the present study showed that Iranian women are more likely to engage in housework and occupational activities rather than regular planned PA [1, 35], which was similar to Hispanic women behaviors [36]. It has been reported that among Iranian women the family’s needs are prioritized above their needs, so that emphasis is placed on the family higher than the individual. In Iranian culture, dedication to family is the driving force with which most of women schedule their daily activities. Cultural-specific characteristics regarding PA among Iranian women usually reflect the principle that family duties and occupational activities could preclude any involvement with PA such as exercise and leisure time activity [1, 35].

The results from testing the Path model showed acceptable fit between the data and the constructs of the HPM model, explain all the significant pathways were in the expected direction. In addition, testing the path model helped to elucidate the effects of prior behaviors resulting from indirect pathways through cognitions constructs, interpersonal influences and commitment to plan. Several previous studies, in which researchers examined only the direct routes of prior behaviors on health behaviors, failed to observe such an association [27, 37, 38].

Prior PA was divided into four categories in present study: the form of transportation, work-related duties, recreational activity and regular physical activity during week. Most of participants reported that they used public transportation or their own cars everywhere. There was little mention that they walk because of shortage of suitable street and sidewalk in city. This result was inconsistent with other studies among European women who reported walking, and for many of the female populations was the most preferred type of PA [36, 39, 40] as well as represented a lifetime of habit; perhaps because walking were more available than other types of PA and provided an opportunity for those with limitations to participate in regular PA [36, 39]. All participants indicated performing PA in the family home every day (e.g., household/yard activities). There was also little report of childhood recreational PA, although a few women reported that they had engaged in recreational activities like swimming and cycling. This finding was consistence to previous studies in Iran that noted Iranian women have little discretionary personal time to engage recreational activities, and usually prefer sedentary leisure time activities as a form of entertainment such as watching television novellas [1, 5].

Although, available health promotion approaches have recently emphasized the benefits of PA, a stronger focus should be placed on promoting women’s beliefs about their own self-efficacy to enhance their confidence in overcoming obstacles to participate in regular physical activities [36, 39]. In this study, self-efficacy was one of the significant predictor that showed a relatively significant proportion of the total effects in PA (16.1%), which was consistent with the Pender’s HPM and previous studies in Iran [13, 41]. Despite significant negative effects of interpersonal influences on self-efficacy, this construct has emerged as a significant predictor because most of participants (64%) possessed a positive sense and belief concerning the benefits of PA. It was also evident the path from feeling to perceived benefit were significant in model testing. A number of studies have reported that a high positive sense of self-efficacy is more likely to promote individual’s confidence regarding personal goals, such as PA [12, 13]. Although we found most of participant’s believed in the benefits of PA, but, they could not actually perform PA on a regular basis because of the various barriers that they cannot be overcome. The majority of participants (85%) reported cultural and social factors (e.g., their traditional role at home, especially responsibility for their spouse and children, spouse opposition due to fear from harassment), employment status, and religion effects (hijab bans) were main barriers that may have affected a woman’s ability, or willingness, to be involved in scheduled programs. This result was consistent with other studies among women who live in Iran [11, 13]. As matter of fact, Iranian women need to learn skills to better understand how setting realistic goals to increase their time for appropriate PA per day or increase small changes that could be realistically fit into their particular lifestyle and daily schedule even in conflicting situations.

Among all the constructs in this study, interpersonal influences were the strongest predictor with negative direct effects on the level of PA that was consistent with Pender’s HPM [26]. There are several possible explanations for the results of a significant and negative relationship between interpersonal influences and PA. First, most of eligible participants (75%) reported weak communication and social network to share attitudes and experiences toward PA behaviors. According to sociological theory, social media styles are directly or indirectly associated with personal responsibility and dedication that impact behavior and social norms [42, 43]. A second finding, we found that most women did not received sufficient support from their family (brother, sister, husband, parent) to undertake PA. This could be due to the cultural factors and lifestyle change in Iran. In some Iranian communities, women are encouraged to be involved in activities for the benefit of the family and not for individual benefit [43, 44]. This present work lends some support to the results of an American survey carried out in an Hispanic population, they posits that within Hispanic cultures, greater emphasis is placed on interpersonal influences and if certain behaviors do not required for the family, then it is not important to them [36]. Further, most Iranian family members and friends holding jobs outside the home and have less time to be active with participants in regular PA or encourage them to use healthy diet. Several studies have reported that people are more likely to perform healthy behaviors when they feel their relatives and friends encourage them frequently to perform those behaviors [1, 5, 23]. Third potential explanation for significant negative effects of interpersonal influences on participation in healthy behaviors is probably due to high women responsibility for taking care of their children, therefore, these women lack of adequate time to involve in scheduled programs. This finding is consistent with other previous studies targeted at HPM findings in Middle East countries [1], Hispanic [45] and Muslim women population [11, 13].

In the present study, the commitment to plan PA emerged as the strongest direct predictor or path of health behaviors when mediates the effects of the other constructs on healthy behaviors, particularly self-efficacy, perceived barriers and interpersonal influences. This finding suggest that those participants who have a higher self-efficacy and perceived barriers show more intention and persistent to engage in PA in the face of obstacles, and have a higher commitment. This finding is consistent with Pender study and other same studies that indicate individuals with higher self-efficacy scores will have a higher level of commitment to PA [22, 23, 46].

Our hypothesis was derived from Pender’s theory that suggests that all cognitive constructs and situational factors influence health-promoting behavior, whereas in this study, perceived benefit, activity related affects and situational influence did not emerge as significant predictors of PA. When all the constructs were included in the Path modeling, the impact of all variables on PA behaviors were tested while controlling for the others to determine the effect of each variables beyond and above the effects of the other variables [23, 46, 47]. Therefore, further studies must be considered to measure the effects of these constructs on commitment in the Iranian population.

This study is subject to some limitations. First, the use of a self-reported questionnaire, may lead to bias. A second limitation is the explanation of causal relationships among variables was limited because of the cross-sectional design of this work. Our participants were selected from one city, so our results reported may not be generalizable to the entire women population in Iran. Third limitation is that the temporal criterion of causality in this study may not be met because of the cross-sectional data. Therefore, future testing of the Pender’s Health Promotion Model with longitudinal data would be useful to better understand the most effective strategies to promote physical activity behaviors and individual’s education program among women population.

Despite these limitations, this study also has several strengths. This study is one of the first to generate knowledge related to identifying the potential determinates or predictors of PA in Iranian women, using Pender’s HPM constructs. Testing the application of the heretical model in different population would be practical for public health educators, policy makers and health promoters in community health practice to better understand women’s needs as well as the critical role of culturally based preventive strategies. Furthermore, we explored how these factors change behavior outcomes and supports people‘s ability to commit to behaviors that may help to better understand modifiable element between individual’s prior behaviors and behaviors outcomes. The findings of present study showed the existence of mutual relationship among cognitive variables that indirectly affect health promotion behaviors. Thus, we suggest further research is required to evaluate the nature of this relationship in greater detail, as has been conducted in a few other investigations. Likewise, the results of this work can serve as a guide to developing educational intervention programs to facilitate a physically active lifestyle, particularly in Muslim country.

## Conclusion

In conclusion, the regression model showed that education attainment, family income, employment status and number of children could be significant predictors of PA behaviors. Likewise, this study highlighted a significant effect of an individual’s prior behaviors, perceived barriers, self-efficacy and interpersonal influences, and low effect of perceived barriers/benefit, feeling, and situational influences on strengthening level of PA and commitment to PA among Iranian Women. Furthermore, our finding showed that most of participants (77% <) were not undertaking the recommended amounts of PA. Reports of low PA exceeded local and global reports and were mainly associated with to cultural and social factors (e.g., insufficient support from their family and their traditional role at home), and long working hours in the private or government sectors. Since, there is no current national program for PA promoting in Iran, therefore, we recommend launching a work/home place health program to promote PA as a viable public health initiative.

## Supplementary Information

**Additional file 1.**

**Additional file 2.**

## Data Availability

The data generated or analyzed during this study is available from the corresponding author on reasonable request.

## Abbreviations

HPM: Health promotion model
PA: Physical activity
