# Supplementary figures and images for: Predictors of physical activity among women in Bojnourd, north east of Iran: Pender’s health promotion model
Source: Arch Public Health. 2021 Oct 14;79:178. doi: 10.1186/s13690-021-00698-x (PMC8518221; doi:10.1186/s13690-021-00698-x)

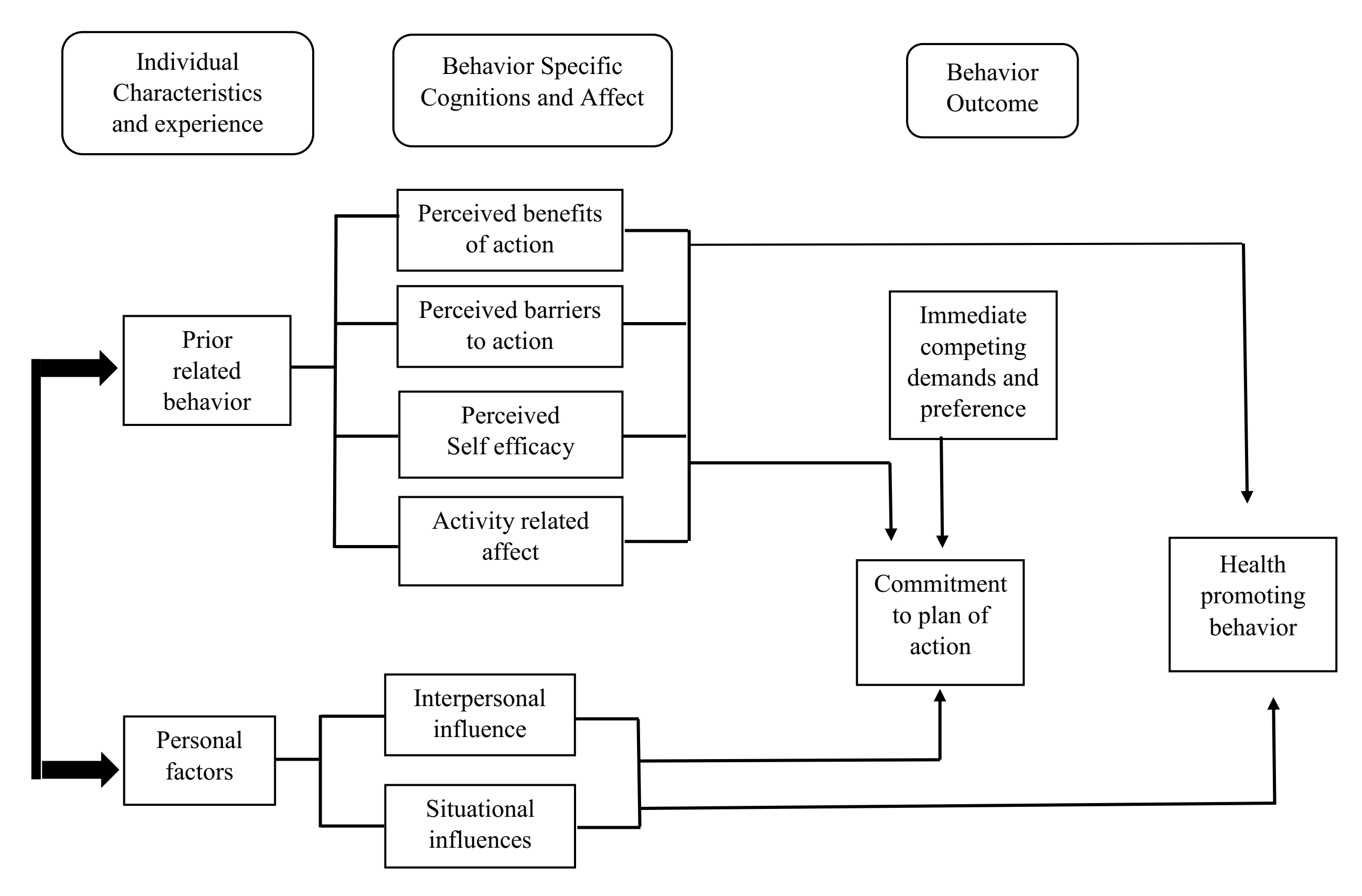


Figure 1s: Pender Health Promotion Model [21]

Supplement: Supplementary file 1 — Additional file 1. [file 13690_2021_698_MOESM1_ESM.docx]
